# Gut Microbiome in Colorectal Cancer: Clinical Diagnosis and Treatment

**DOI:** 10.1016/j.gpb.2022.07.002

**Published:** 2022-07-30

**Authors:** Yali Liu, Harry Cheuk-Hay Lau, Wing Yin Cheng, Jun Yu

**Affiliations:** Institute of Digestive Disease and The Department of Medicine and Therapeutics, State Key Laboratory of Digestive Disease, Li Ka Shing Institute of Health Sciences, CUHK Shenzhen Research Institute, The Chinese University of Hong Kong, Hong Kong Special Administrative Region 999077, China

**Keywords:** Gut microbiome, Colorectal cancer, Diagnostic biomarker, Immunotherapy, Chemotherapy

## Abstract

**Colorectal cancer** (CRC) is one of the most frequently diagnosed cancers and the leading cause of cancer-associated deaths. Epidemiological studies have shown that both genetic and environmental risk factors contribute to the development of CRC. Several metagenomic studies of CRC have identified gut dysbiosis as a fundamental risk factor in the evolution of colorectal malignancy. Although enormous efforts and substantial progresses have been made in understanding the relationship between human **gut microbiome** and CRC, the precise mechanisms involved remain elusive. Recent data have shown a direct causative role of the gut microbiome in DNA damage, inflammation, and drug resistance in CRC, suggesting that modulation of gut microbiome could act as a powerful tool in CRC prevention and therapy. Here, we provide an overview of the relationship between gut microbiome and CRC, and explore relevant mechanisms of colorectal tumorigenesis. We next highlight the potential of bacterial species as clinical biomarkers, as well as their roles in therapeutic response. Factors limiting the clinical translation of gut microbiome and strategies for resolving current challenges are further discussed.

## Introduction

Colorectal cancer (CRC) accounts for approximately 10% of all cancers diagnosed worldwide, with almost 700,000 associated deaths every year, making it the world’s third most deadly cancer [1]. CRC progresses from polyp, adenoma, and to malignant tumor, and this transformation, which is influenced by various genetic and environmental factors, may take many years to complete. Evidence from twin and family studies reveals that the heritability of CRC is only 12%–35%, reflecting the importance of environmental factors in the development of CRC [2]. In particular, Western diets and lifestyles have been associated with CRC in a microbiome-modulation way [3], [4].

The bacteria residing in the large intestine constantly interact with the colonic epithelial cells and other microbes, and modulate physiological processes such as energy exchange and host immunity [5], [6]. Owing to the close link between the gut microbiome and normal physiology, dysbiosis in the gut microbiome leads to various diseases, including cancer. In particular, the enrichment of pathogenic microbes in a dysbiotic microbiome has been closely associated with cancer development and progression. For instance, *Helicobacter pylori* is infamous for its capability to cause chronic gastric mucosal inflammation and genetic instability, subsequently contributing to gastric tumorigenesis [7]. Although there is currently no direct evidence that a specific bacterial species can induce CRC, several microbes, including *Bacteroides fragilis*, *pks^+^ Escherichia coli*, and *Fusobacterium nucleatum*, have demonstrated their capabilities to promote CRC development [8].

In humans, the pathological imbalance of the gut microbiome is present in CRC patients, which has been shown to be strongly associated with cancer initiation and progression [9]. Microbiome alterations occurring in the early stage of CRC also highlight the potential of using specific bacterial species as non-invasive diagnostic biomarkers for CRC [10]. Common screening tests for CRC, such as guaiac fecal occult blood tests (gFOBTs) and fecal immunochemical tests (FITs), have low sensitivity for early and advanced neoplasia [11], while recent studies have shown that the combination of the fecal microbiome data and the standard gFOBTs or FITs leads to marked improvements in CRC detection [12]. Moreover, the explosive growth of pathogens, such as *F. nucleatum* [13], along with the accumulation of toxins in the large intestine, influences not only the development of CRC but also the efficacy of immunotherapy and chemotherapy. For example, CRC patients with high intratumoral abundance of *F. nucleatum* are more resistant to oxaliplatin [14]. A preclinical study using a xenograft animal model showed that *F. nucleatum* activates autophagy of cancer cells through toll-like receptor (TLR)-4 and myeloid differentiation primary response 88 (MYD88) signaling pathway, potentiating CRC resistance to oxaliplatin and 5-fluorouracil (5-Fu) regimens [14]. Thus, there is a growing interest in identifying microbial markers for cancer diagnosis and developing microbial-based adjuvant therapies to enhance cancer treatment efficacy [15]. This review summarizes the relationship between the gut microbiome and CRC as shown by existing evidence, including the pathophysiological mechanisms of gut dysbiosis-related CRC, and the impact of microbiome disorders on the diagnosis and clinical treatment of CRC.

## Correlation between the gut microbiome and CRC

High-throughput sequencing technology has become a popular approach in microbial analysis. The metagenomic data collected from fecal and tissue samples not only provide the microbial profile in humans with high resolution at species or even strain level, but also reveal the functions of the gut microbiome and its interactions with the human host. In particular, the gut microbial imbalance, as supported by numerous basic and clinical studies, is one of the main characteristics of CRC. For instance, the abundances of pro-carcinogenic taxa (*B. fragilis*, *Enterococcus faecalis*, *E. coli*, *F. nucleatum*, *Peptostreptococcus anaerobius*, *Porphyromonas*, and *Micromonas parvum*) increase significantly in CRC patients, while some potentially protective taxa (*Clostridium butyicum*, *Roseburia*, and *Bifidobacterium*) show reduced abundances [16]. Analysis of tissue biopsies collected at different stages of CRC also highlights the role of gut dysbiosis in adenoma, suggesting the functional importance of the gut microbiome in the initiation and development of CRC [17]. The impact of the gut microbiome on CRC is further supported by the observation that germ-free mice and conventional mice treated with azoxymethane (AOM) generated more polyps and showed higher levels of intestinal dysplasia when fed with feces of CRC patients [18].

## Mechanisms of gut microbiome involved in CRC development

Recently, several pathogens, such as *F. nucleatum*, *B. fragilis*, and *P*. *anaerobius*, have been reported to contribute to colorectal tumorigenesis via diverse mechanisms, including promotion of inflammation, bacterial adhesion to host cells, and toxin production [8]. Here, we listed several distinguished mechanisms utilized by gut bacteria to promote the progression of CRC.

### Genotoxic effects of pathogenic bacteria

Several pathogenic bacteria can directly interact with host cells and induce tumorigenesis through DNA damage and pro-inflammatory effect. For example, *F. nucleatum* adheres to N-acetyl-D-galactosamine (Gal-GalNAc)-expressing CRC cells by its surface protein Fap2, facilitating its colonization and enrichment in tumor tissue [19]. Similarly, *P. anaerobius* directly binds to the surface receptor integrin α2/β1 (ITGA2/ITGB1) of intestinal epithelial cells through its surface protein putative cell wall binding repeat 2 (PCWBR2) to activate the tumor-promoting pathway PI3K-Akt-NF-κB, resulting in hyperproliferation of cancer cells [20], [21]. Apart from gut bacteria themselves, bacteria-derived virulence factors also modulate the transformation of normal colonic epithelial cells to tumor cells. *F*. *nucleatum* secrets adhesin FadA, which can bind to E-cadherin to activate β-catenin signaling, promoting inflammatory and oncogenic responses. *E*. *coli* with the *pks* virulence island is another gut bacterium that is enriched in human CRC tissues and has been shown to enhance tumorigenesis in preclinical CRC models. *pks^+^ E. coli* produces the cytolethal distending toxin, a group of heat-labile protein exotoxins that can infect intestinal mucosa, induce inflammation, and increase the frequency of host cell mutations [22]. *pks^+^ E. coli* also encodes the polypeptide colibactin. Infecting eukaryotic cells with colibactin results in double-strand DNA breaks, eukaryotic cell cycle arrest, and chromosome aberrations, thereby promoting colorectal tumorigenesis [23]. Similarly, enterotoxin-producing *B. fragilis* secretes a zinc-dependent metalloprotease known as *B. fragilis* enterotoxin (BFT). BFT causes inflammation in preclinical CRC models, increasing intestinal permeability and preceding the process of pathogen transmigration. BFT-mediated cleavage of E-cadherin also initiates oncogenic responses through activating WNT signaling pathway and stimulating the release of β-catenin to activate the expression of genes such as *CCND1* or *MYC* [24].

### Immune modulation by the gut microbiome

Gut microbes modulate the inflammatory processes in the intestine and stimulate the development and maturation of the host immune system. Accumulating evidence has shown that the gut microbiome influences CRC initiation and progression by chronic infection and inflammation. A colitis-associated CRC mouse model has shown that inflammation-induced changes in the microbial composition promote colorectal tumorigenesis [25]. Specifically, chronic inflammation creates a favorable environment for bacteria with genomic toxicity, such as *pks*^+^ *E. coli*, which adheres to the colonic mucosa and induces host DNA damage, promoting CRC in AOM-treated mice. In contrast, inflammation along in the absence of *pks*^+^ *E. coli* is insufficient to induce CRC [26]. Furthermore, transplanting feces of CRC patients into germ-free mice increases tissue inflammation and the expression of pro-inflammatory genes [18]. On the contrary, fecal microbiota transplantation (FMT) from long-term survivors of CRC boosts the immune response and limits tumor growth in mouse models by altering the tumor microbiome [27]. Mechanistically, the gut microbiome releases chemokines, recruiting immune cells to tumors. Bacteria-derived lipopolysaccharide (LPS) stimulates CC chemokine ligand 2 (CCL2) expression in colonic epithelial cells and induces the accumulation of monocyte-like macrophage (MLM). LPS further stimulates interleukin (IL)-1β production from MLM, inducing the activation of IL-17-producing T-helper (TH) cells and generating a precancerous inflammatory milieu to facilitate tumourigenesis [28].

Microbial sensing by innate immune receptor signaling also results in tumorigenesis. Pathogenic microbes can be recognized by the pattern recognition receptors (PRRs) of the host, including TLRs and nucleotide-binding oligomerization domain (NOD)-like receptors (NLRs) [29]. Bacteria-derived signals identified by PRRs can activate downstream inflammatory signaling pathways, including nuclear factor kappa-light-chain-enhancer of activated B cells (NF-κB), mitogen-activated protein kinase (MAPK), and signal transducer and activator of transcription 3 (STAT3) which are all important pathways that bridge inflammation with cancer [30]. *P*. *anaerobius* specifically activates TLRs, which mediate the increased expression level of reactive oxygen species (ROS), leading to dysplasia in the colon of AOM-treated mice [21]. MYD88 is another key downstream molecule of TLR activation and contributes to the development of CRC. Pathogenic bacteria in the tumor can activate TLR4 of mesenchymal cells and the MYD88 pathway, releasing the inflammatory factor IL-23, which in turn activates IL-17A, IL-6, and IL-22 to promote the development of CRC [31]. In addition to TLRs, gut microbes also modulate inflammatory responses through NLRs, another key member of the PRR superfamily. With the ability to recognize the intracellular fragments of bacterial peptidoglycan, NOD1 and NOD2, central members of NLRs, have been reported to act as regulators of the innate and adaptive immune responses [32]. Intestinal commensal bacteria induce the maturation of the intestinal immune system through NOD1 signals, while NOD1 deficiency leads to epithelial cell apoptosis and increases intestinal permeability, promoting CRC in mice [33]. Meanwhile, NOD2-deficient-related gut dysbiosis has also been shown to increase susceptibility to CRC [34]. Previous studies have shown that NOD1 plays an important role in the induction of innate immune responses and inflammatory cues when sensing invading bacteria. However, the inflammatory responses also may cause detrimental effects on the progression of CRC. Emerging evidence has shown that NOD1 is highly expressed in human CRC, of which NOD1 activation augments CRC cell adhesion, migration, and metastasis [35]. In addition to the recognition of intestinal microbes by PRRs, several pathogenic bacteria can directly bind to the host cell receptors. For example, the Fap2 protein of *F. nucleatum* binds to the immunosuppressive receptor TIGIT to inhibit cytotoxic effects of T cells and nature killer cells on tumors. *F*. *nucleatum* also produces FadA adhesin to activate the oncogenic WNT/β-catenin pathway and the pro-inflammatory NF-κB pathway, contributing to CRC development [36], [37].

### Microbial metabolome and CRC

In addition to the pro-carcinogenic activities of specific pathogens, the gut microbiome produces metabolites to influence the development and progression of CRC. Gut microbes participate in fermentation and produce secondary metabolites such as short-chain fatty acids (SCFAs) and indole compounds, as well as bile acids which can promote the formation of adenoma by inducing DNA damage, pro-inflammatory effects, cell proliferation, and apoptosis [38]. Recent research has shown that the levels of microbial metabolites, including branched-chain amino acids, phenylalanine, and bile acids, are significantly elevated in multiple polypoid adenomas and intramucosal carcinomas, suggesting the potential of microbial metabolites as markers for early CRC screening [39]. Bile acids are a type of steroid acids synthesized in the liver and converted into several different secondary bile acids by bacteria in the intestines [40]. The main secondary bile acids include taurodeoxycholic acid, lithocholic acid, and deoxycholic acid (DCA). In particular, numerous studies have illustrated the association of DCA with CRC. For instance, DCA levels in feces, intestine, and serum are increased in individuals at high risk for CRC and in CRC patients compared to healthy controls [41]. AOM-treated mice also exhibit the increased formation of colon adenomas upon receiving different concentrations of DCA [42]. The molecular mechanisms that mediate the cytotoxic effects of DCA are complex. Preclinical experiments have shown that DCA blocks the activation of the NF-κB signaling pathway and the nuclear translocation of nuclear transcription factor RelA, inducing inflammation and tumorigenesis in the gut [43]. DCA also includes DNA damage to host cells that is induced by ROS generation and β-catenin signaling activation, thereby contributing to CRC development. Besides, DCA can also modulate the gut microbiome to a dysbiotic composition, which mediates tumor-promoting activities in the gut. DCA arises from cholic acid (CA), and supplementing CA to rats increases DCA concentrations in cecum to 0.98–2.55 mmol/l [44]. Recent studies suggest that supplementation with different concentrations of CA causes enrichment in classes Clostridia and Erysipelotrichi [44]. Despite showing no tumor formation in the colon, the increased production of DCA by bacterial 7α-dehydroxylation reaction is also correlated with the increased risk of CRC [44]. Taken together, the gut microbiome regulates the composition of the bile acid pool, resulting in the accumulation of toxic secondary bile acids. In turn, bile acids can modulate the gut microbiome, further contributing to the development of CRC.

On the contrary, some bacterial metabolites have protective and anti-tumorigenic effects against CRC. SCFAs, including propionate and butyrate, are the main metabolites produced by the fermentation of indigestible carbohydrates such as fiber and resistant starch [45]. As an anti-inflammatory molecule, butyric acid inhibits histone deacetylases in colonocytes and immune cells, promoting hyperacetylation of specific transcription factors and proteins involved in signal transduction. This, in turn, leads to down-regulation of pro-inflammatory factors, inhibition of cell proliferation, and selective induction of apoptosis in CRC cells [46]. Other important anti-tumorigenic effects of butyrate include inhibiting angiogenesis and suppressing the proliferation of gut pathogenic bacteria [47]. A preclinical study has shown that butyrate activates peroxisome proliferator-activated receptor gamma (PPAR-γ) and further drives the energy metabolism of colonocytes toward β-oxidation, maintaining the hypoxic environment in the intestinal lumen. The epithelial PPAR-γ signaling also limits luminal nitrate availability by inhibiting *NOS2* expression (encoding inducible nitric oxide synthase). These effects of butyrate lead to the limitation in the excessive proliferation of potentially pathogenic *E. coli* and *Salmonella* in the colon [48]. Moreover, in a healthy or precancerous state, butyrate can act as the major energy source for colonocytes to promote their proliferation and epithelial growth, thereby increasing the crypt depth, thickening the mucosa, and reinforcing the intestinal barrier, which all can contribute to CRC prevention [49]. Indole-derived metabolites produced by the metabolism of tryptophan by *Clostridium* spp. and *Bacteroides* spp., which act as endogenous ligands for the aromatic hydrocarbon receptor (AHR), also show a protective effect against CRC. Indole-3-aldehyde can affect the systemic immune status through the AHR/IL-22 axis to inhibit the occurrence of inflammatory CRC [50].

## Gut microbial markers for the diagnosis of CRC

**Figure 1:**
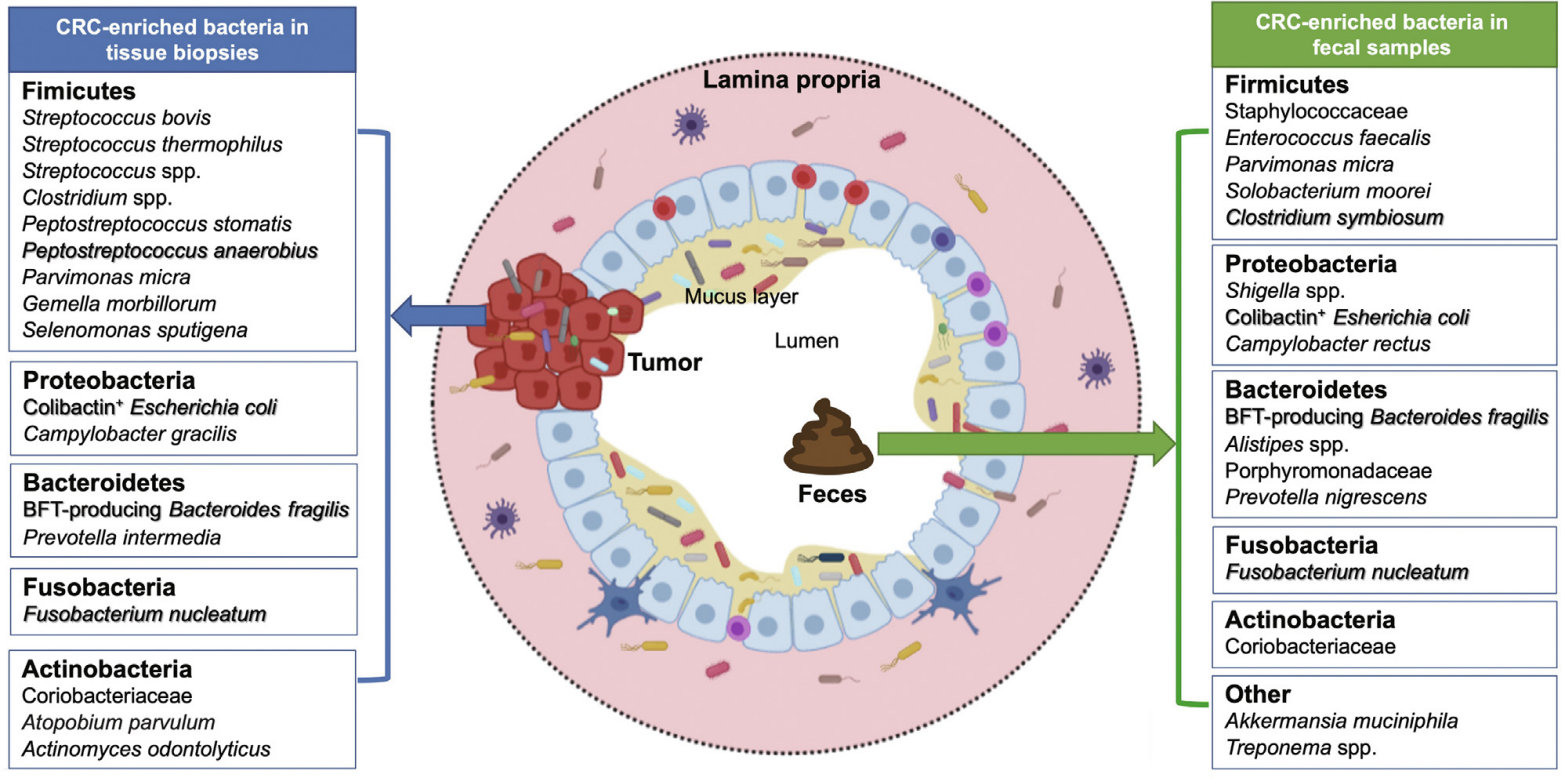
**Enriched bacterial species in CRC** Several bacterial species are significantly enriched in tissue biopsies or fecal samples of patients with CRC. These bacteria can potentially serve as CRC biomarkers. For example, combining the detection of fecal *Fusobacterium nucleam* and *Clostridium symbiosum* with FIT has improved the diagnostic performance of advanced adenoma and CRC [55], [95]. The sensitivity of FIT for advanced adenoma can also be enhanced by detecting the enrichment of a group of genera, including *Fusobacterium*, *Peptostreptococcus*, *Porphyromonas*, *Prevotella*, *Parvimonas*, *Bacteroides*, and *Gemella*[96]. Well-studied CRC-enriched bacteria are highlighted in bold and shadow. CRC, colorectal cancer; FIT, fecal immunochemical test.

Abnormality in the composition of the gut microbiome has been implicated in the initiation and progression of CRC, indicating that an altered gut microbiome is an important etiologic factor in CRC development. Moreover, the analysis of microbial communities in fecal and mucosal samples has revealed that specific changes in the gut microbiome are associated with distinct stages of CRC [51], [52], [53]. These specific microbial markers distinguish CRC from healthy controls, indicating the diagnostic potential of gut microbes in CRC detection (Figure 1). Distinguishing candidate microbes for predicting CRC is challenging, given the high inter-individual variability of microbiome composition, which is attributed to the disparities in sex, age, diet, lifestyle, genetic background, and medication use. Nevertheless, notable progress in this field has been made. Several studies were able to determine correlations between fecal microbial dysbiosis and CRC diagnosis. In 2014, Zackular et al*.* [54] characterized the fecal microbiomes of 30 CRC patients, 30 colonic adenoma patients, and 30 healthy controls to establish a classification model for CRC diagnosis. By combining the microbiome data with known clinical risk factors (*e.g.*, body-mass index, age, and race), the authors found that the microbiome could significantly improve the ability to predict CRC compared to risk factors along. Notably, this study was based on 16S ribosomal RNA gene analysis and did not perform any independent validation [54].

In 2015, our team performed metagenome-wide association studies on fecal samples of 74 patients with CRC and 54 healthy controls in the Chinese cohort [10]. We discovered two new species associated with CRC, *Parvimonas micra* and *Solobacterium moorei*, as well as 20 gene markers that can significantly differentiate CRC-associated and control microbiomes. Importantly, 4 gene markers were further validated in published independent cohorts from French and Austrian, suggesting that signatures of CRC-associated microbial dysbiosis could have universal features. We further used 2 microbial gene markers (the butyryl-CoA dehydrogenase gene of *F. nucleatum*, and the RNA polymerase β subunit gene of *P. micra*) to separate CRC microbiomes from controls and achieved high accuracy [area under the curve (AUC) = 0.84] [10]. This study provided a proof-of-principle that establishing diagnostic test using fecal microbial gene markers to identify patients with CRC may indeed be possible. Then in 2017, our team evaluated fecal microbial markers for clinical use in detecting CRC and advanced adenoma [55]. We found that combining the abundance of *F. nucleatum* with FIT can increase the sensitivity of FIT detection from 73.1% to 92.3%. We also identified that the combination of *F. nucleatum*, *Bacteroides clarus*, *Roseburia intestinalis*, and *Clostridium hathewayi* with FIT could also improve sensitivity and specificity for the diagnosis of CRC and colon adenoma. These approaches offer significant promise for incorporating microbial biomarkers into routine clinical practice to aid diagnosis [55]. Of note, although large cohort studies have identified associations and potential for microbes to be used in CRC diagnosis, there are still limitations because most studies focus on the detection of advanced stages of CRC. Also, there is a limited agreement in the taxa reported because different populations can have distinct microbial community structures.

Recently, researchers have begun to clarify the changes in the gut microbiome in the early stages of CRC, such as polyps, adenomas, and other precancerous colorectal lesions. In 2019, a study from Japan collected fecal samples from 631 participants, including patients with multiple polypoid adenomas, intramucosal carcinoma (stage 0 and stages I–IV), and healthy controls [56]. Metagenomic and metabolomic analyses were used to assess taxonomic and functional characteristics of the gut microbiome and metabolites. This study revealed that *F. nucleatum*, *Atopobium parvulum*, and *Actinomyces odontolyticus* were significantly enriched in multiple polypoid adenomas and/or in stage 0 CRC, suggesting that these bacteria are useful for CRC diagnosis in the early stage [56]. Apart from the microbial profile, the fecal branched-chain amino acids, phenylalanine, and DCA were also identified as the best-scoring markers to distinguish stage 0 CRC cases from healthy controls [56].

Fungi and viruses are important components of the gut microbiome and also have potential as biomarkers of CRC. In a metagenome-wide association study involving 184 patients with CRC, 197 patients with adenoma, and 204 control subjects, the homeostasis of the gut fungal community was found to be destroyed in CRC. Specifically, the Basidiomycota:Ascomycota ratio is higher in CRC patients compared to healthy controls, with enrichment of class Malasseziomycetes and depletion of classes Saccharomycetes and Pneumocystidomycetes. Fourteen fungal biomarkers were then identified with great performance to distinguish CRC from healthy controls (AUC = 0.93), and distinguish early-stage CRC from healthy controls (AUC = 0.91), which were further validated in an independent cohort. This study therefore elucidated for the first time that the enteric fungal profile is associated with CRC, and fungal markers can be potentially used for CRC detection [57]. Similarly, the analysis of viral profiles in fecal samples showed that the diversity of gut phages increased significantly in CRC. The abundances of 22 viral biomarkers could distinguish CRC patients from healthy controls with an AUC of 0.93 [57], [58].

## Gut microbes and CRC prevention and therapy

Given the crucial roles of gut microbes in CRC, numerous investigations which aim to target the gut microbiome to reduce CRC risk have been conducted. Diets and lifestyles can change the gut microbiome and associated metabolites to promote CRC. An unhealthy diet, such as excess fat intake, has been reported to accelerate colorectal tumorigenesis by inducing gut dysbiosis with the enrichment of pathogenic bacteria and the accumulation of the harmful metabolite lysophosphatidic acid [59]. Conversely, high fiber intake is associated with increased levels of SCFAs and a higher abundance of SCFA-producing bacteria (*e.g.*, *Eubacterium rectale* and *Clostridium symbiosum*), which modulate host immunity and suppress inflammation to prevent CRC [60]. Meanwhile, a previous meta-analysis has also reported that individuals with frequent physical activity have a reduced risk of CRC [61]. Exercise can increase the microbiome diversity, along with the enrichment of SCFA-producing bacteria [62], [63], [64]. Of note, the optimal type, intensity, and duration of exercise for CRC prevention are underdetermined. Extensive preclinical studies and clinical trials are needed to decipher the mechanistic roles and effectiveness of microbiome modulation to prevent CRC development.

**Figure 2:**
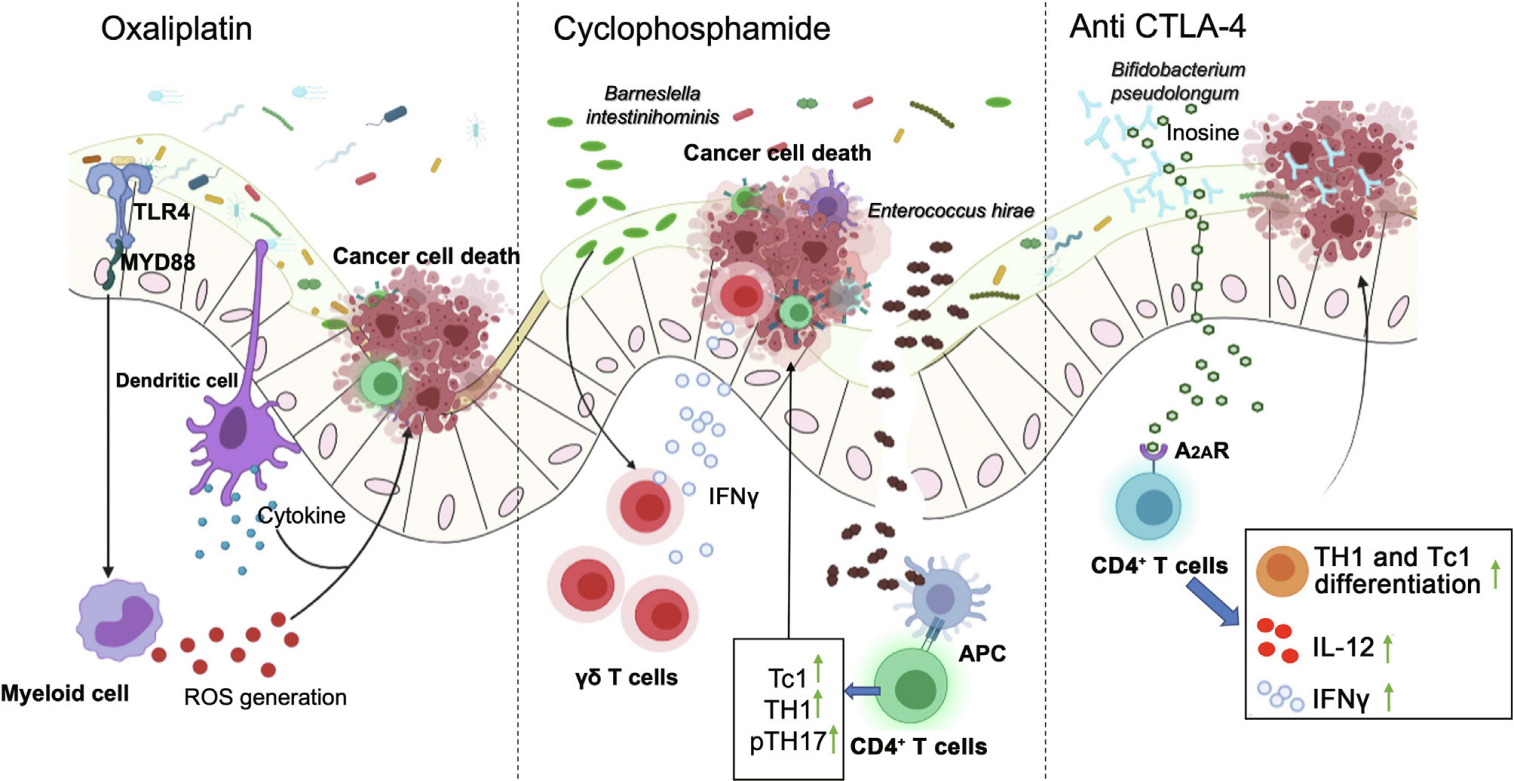
**Influence of the gut microbiome on CRC chemotherapy and immunotherapy** Gut commercial microbes mediate the response of cancer cells to the chemotherapeutic drug oxaliplatin by modulating the functions of myeloid-derived cells in the tumor microenvironment [68]. *Barneslella intestinihominis* and *Enterococcus hirae* can be translocated to lymphoid tissues, facilitating immunomodulatory effects induced by another chemotherapeutic drug, cyclophosphamide [71]. *Bifidobacterium pseudolongum* produces the metabolite inosine to enhance the anti-CTLA-4 immunotherapeutic response through activating A_2A_R expressed in antitumor T cells [84]. A_2A_R, type 2a adenosine receptor; APC, antigen-presenting cell; CTLA-4, cytotoxic T lymphocyte-associated antigen-4; IFNγ, interferon-gamma; IL-12, interleukin-12; MYD88, myeloid differentiation primary response 88; pTH17, pathogenic T helper 17 cell; ROS, reactive oxygen species; Tc1, type 1 CD8^+^ T cell; TH1, T helper 1 cell; TLR4, toll-like receptor 4.

On the other hand, the gut microbiome can modulate the response to a variety of chemotherapeutic drugs and immune checkpoint blockers, including toxicity and efficacy, through drug metabolism, immune regulation, and other mechanisms (Figure 2; Table 1).

**Table 1:** **Effects of the gut microbiome on CRC treatment**

**Bacterial species**	**Role**	**Effect on therapy**	**Mechanism**	**Ref.**
*Fusobacterium nucleatum*	Detrimental	Promote oxaliplatin resistance	Activate TLR4/MYD88 to induce autophagy	[14]
Gammaproteobacteria	Detrimental	Cause gemcitabine resistance	Mediate intratumoral gemcitabine deamination	[97]
*Escherichia coli*; *Clostridium difficile*	Detrimental	Induce side effects of irinotecan	Produce β-glucuronidases to reactivate toxic SN-38	[98]
*Lactobacillus paracasei* spp. NTU 101	Beneficial	Sensitize 5-Fu	Produce metabolites to increase antitumor effects	[99]
*Lactobacillus plantarum*	Beneficial	Sensitize 5-Fu	Promote SMCT1/butyrate-mediated tumor suppression	[100]
*Lactobacillus casei*; *Lactobacillus rhamnosus*	Beneficial	Alleviate FOLFOX-induced mucosal damage	Down-regulate NF-κB pathway, TNF-α, and IL-6; reduce apoptosis	[101]
*Saccharomyces boulardii*	Beneficial	Alleviate irinotecan-induced mucosal damage	N/A	[102]
*Streptococcus* spp.	Beneficial	Alleviate irinotecan-induced diarrhea and toxicity	N/A	[76]
*Bifidobacterium bifidum*	Beneficial	Enhance 5-Fu efficacy	N/A	[103]
*Bacteroides thetaiotaomicron*; *Bacteroides fragilis*	Beneficial	Enhance anti-CTLA-4 efficacy	Activate IL-12-dependent Th1 immune response	[104]
*Bifidobacterium pseudolongum*	Beneficial	Enhance anti-PD-1 efficacy	Produce metabolite inosine to improve antitumor immunity by activating the A_2A_R in T cells	[84]
*Bifidobacterium breve*	Beneficial	Enhance anti-PD-1 efficacy	Promote DC maturation and CD8^+^ T cell activation	[105]
*Lactobacillus acidophilus*	Beneficial	Enhance anti-CTLA-4 efficacy	Inhibit M2 macrophage, Treg; stimulate effector memory T cells and CD8^+^ T cells	[106]
*Fusobacterium nucleatum*	Beneficial	Enhance anti-PD-L1 efficacy	Activate STING signaling; recruit IFNγ^+^ CD8^+^ tumor-infiltrating lymphocytes	[107]
*Lactobacillus rhamnosus* spp. ATCC 7469	Beneficial	Enhance the efficacy of radiotherapy	Produce EPS to inhibit p38 MAPK and NF-κB signaling	[108]

*Note*: N/A, not available; 5-Fu, 5-fluorouracil; A_2A_R, type 2a adenosine receptor; APC, antigen-presenting cell; CTLA-4, cytotoxic T lymphocyte-associated antigen-4; DC, dendritic cell; EPS, exopolysaccharide; IFNγ, interferon-gamma; IL, interleukin; MAPK, mitogen activated protein kinase; MYD88, myeloid differentiation primary response 88; NF-κB, nuclear factor kappa-light-chain-enhancer of activated B cells; PD-1, programmed cell death-1; PD-L1, PD-1 ligand-1; SMCT1, sodium-coupled monocarboxylate transporter 1; Th1, T helper 1 cell; TLR4, toll-like receptor 4; TNF-α, tumor necrosis factor-alpha.

### Gut microbiome in chemotherapy

The chemotherapeutic drugs are a major staple of cancer therapy, which can act on different parts in the growth and proliferation of tumor cells. Commonly used chemotherapeutic drugs include alkylating agents, antimetabolites, antitumor antibiotics, and platinum [65]. The gut microbiome regulates the response to cancer chemotherapy through various mechanisms, such as immune regulation, translocation, and enzymatic degradation. Chemotherapeutic drugs alter the tumor microenvironment and evoke tumor-destructive immune responses through commensal bacteria [65], [66], [67]. For instance, the platinum compounds oxaliplatin and cisplatin cause tumor cytotoxicity by forming platinum DNA adducts and intra-strand cross-links [68]. However, their antitumor effect is attenuated significantly in antibiotic-treated or germ-free mice. Antibiotic treatment reduces the expression of pro-inflammatory genes induced by oxaliplatin, as well as the genes related to monocyte differentiation, activation, and function, suggesting that the microbes play an important role in the antitumor effect of chemotherapeutic drugs. Antibiotic treatment not only attenuates the production of ROS, which is required for oxaliplatin to exhibit genotoxicity in tumor cells, but also hinders ROS production by tumor-infiltrating immune cells [68]. This study suggests that commensal bacteria can affect the type of inflammatory tone required for response to chemotherapy.

The intestinal barrier in cancer patients is greatly damaged. The symbiotic microbiome and pathogenic bacteria can therefore translocate to the pancreatic lymph nodes or distant organs through the impaired barrier, regulating the efficacy of chemotherapeutic drugs via inducing autoimmune effects. For example, cyclophosphamide (CTX) is a widely used antineoplastic agent. However, CTX-induced toxicity is not limited to tumor tissue but also hematopoietic cells and intestinal epithelial cells, leading to alteration in the gut microbiome. Administration of CTX increases the abundance of potentially pathogenic bacteria (*E. coli*, Enterobacteriaceae, *Pseudomonas*, and *Enterococci*) and disrupts the intestinal mucosal barrier, thus facilitating bacterial translocation from the gut to the circulation. Moreover, mouse studies have found that the antitumor effect of CTX on subcutaneous transplantable tumors is dramatically decreased after treatments of broad-spectrum antibiotics and vancomycin, indicating that the gut microbiome plays an essential role in modulating the antitumor effect of CTX. More specifically, CTX promotes the translocation of distinct Gram-positive bacteria, such as *Lactobacillus johnsonii* or *Enterococcus hirae*, into secondary lymphoid organs [69], where these bacteria stimulate the generation of a specific subset of “pathogenic” TH17 (pTH17) cells and the differentiation of naïve CD4^+^ T cells into TH1 and TH17 cells. Oral gavage of *L. johnsonii* and *E. hirae* facilitate reconstitution of the pool of pTH17 cells in the spleen of antibiotic-treated mice, and adoptive transfer of pTH17 cells partially restores the antitumor efficacy of CTX, suggesting that the translocation of a specific set of Gram-positive commensal bacteria is necessary and sufficient to mediate the CTX-driven antitumor immune response [70]. Subsequent studies also found that the Gram-negative *Barnesiella intestinihominis* ameliorates the effects of CTX. The accumulation of this bacterial species in the colon markedly influences the abundance of polyfunctional splenic TH1 and type 1 CD8^+^ T cells and increases the recruitment or proliferation of interferon (IFN)-γ^+^ γδT cells in tumor-infiltrating lymphocytes, behaving as “oncomicrobiotics” with CTX against a wide spectrum of mouse cancers [71]. Thus, these studies support the use of commensal bacteria as an adjuvant to modulate the antitumor effect of chemotherapeutic drugs.

The gut microbiome can also influence the pharmacokinetics and cytotoxicity of anti-CRC drugs. Antitumor drugs can induce changes in the composition and gene expression of the gut microbiome. The altered gut microbes can then contribute to drug efficacy and safety by metabolism. Our team discovered that *Lysinibacillus sphaericus* degrades aspirin and abolishes its preventive effect against the development of colorectal tumors [72]. Aspirin showed a significant antitumor effect in microbiome-depleted mice, but not in mice with the intact microbiome. More specifically, our analysis showed that an intact gut microbiome could induce biotransformation of aspirin. Anaerobic culture of fecal microbiome from antibiotic-naïve mice and metagenomic analysis identified *L. sphaericus* as the prominent microbe involved in the effect of aspirin. The ability of *L. sphaericus* to inhibit the antitumor effect of aspirin and to increase the degradation of aspirin and its active metabolite salicylic acid was confirmed by oral administration of bacteria in germ-free mice. Moreover, aspirin treatment resulted in the accumulation of probiotic bacteria, including *B*. *pseudolongum*, *Bifidobacterium breve*, *Bifidobacterium animalis*, *Lactobacillus reuteri*, *Lactobacillus gasseri*, and *L. johnsonii*, which may contribute to its CRC protective effect. Consistently, colonizing fecal microbiome from aspirin-treated *Apc*^min/+^ mice (a transgenic CRC mouse model) in carcinogen-treated germ-free mice causes a reduction in tumor number and tumor load. These results indicate a direct contribution of the gut microbiome in mediating the CRC chemoprevention of aspirin.

Oral drugs are mostly metabolized and absorbed in the gastrointestinal tract, resulting in a great contribution of bacterial enzymes to the bioavailability of these drugs, while injected drugs can also be exposed to the gut microbiome through biliary excretion. Irinotecan (CPT-11) is a widely used anticancer drug for the treatment of CRC. Irinotecan is converted by hepatic or gastrointestinal carboxylesterases to its active metabolite SN-38, responsible for the strong antitumor activity and cell toxicity, which is then conjugated in the liver by glucuronyltransferase to its inactive form SN-38G before secreted into the gut. However, SN-38G is susceptible to bacterial β-glucuronidase, which can be converted back to SN-38 in the gut, thereby increasing SN-38 in the gut and contributing to toxicity [73]. Irinotecan induces changes in the gut microbes, especially those with β-glucuronidase activity (*E. coli*, *Staphylococcus* spp*.*, *Bacteroides* spp*.*, and *Clostridium* spp.), those that were suggested to inhibit β-glucuronidase activity (*Lactobacillus* spp.), and those with general beneficial effects to the intestine (*Bifidobacterium* spp.). The unbalanced gut microbiome causes the up-regulation of β-glucuronidase activity, resulting in the accumulation of SN-38 in the gut and irinotecan-induced diarrhea [74]. Recent studies show that bacterial β-glucuronidase inhibitors partially alleviate irinotecan-induced tissue damage and resultant diarrhea in mice by blocking the irinotecan-induced bloom of Enterobacteriaceae and increasing epithelial regeneration [75]. Moreover, probiotic use in clinical trials can also reduce gastrointestinal toxicity induced by irinotecan [76]. Indeed, the microbial composition modulates the response and cytotoxicity of anti-CRC drugs by complex mechanisms. It has been reported that *F. nucleatum* can regulate TLR4-mediated pathway activation and MYD88-induced autophagy in tumor cells to cause a weak response to 5-Fu, capecitabine, and oxaliplatin [14], therefore providing a guideline for clinicians to select appropriate therapy for *F. nucleatum*-positive CRC patients.

Preclinical studies have provided ample evidence on the connection between the gut microbiome and chemotherapy. A few clinical trials have also shown that the modulation of microbiome could improve CRC treatment efficacy. A recent randomized double-blinded trial assessed the effect of probiotic consumption with 6 species of *Lactobacillus* and *Bifidobacterium* in 52 CRC patients after surgery [77]. The results showed that the level of pro-inflammatory cytokines was significantly reduced in patients receiving probiotics, suggesting that probiotics have the potential to suppress inflammation associated with CRC. Meanwhile, the effects of probiotics on chemotherapy efficacy were tested in several clinical trials. For instance, supplementation of *Lactobacillus rhamnosus* GG could alleviate toxicity induced by 5-Fu in CRC patients, reducing the incidence of chemotherapy-induced diarrhea and abdominal discomfort [78]. Consumption of the probiotic formula colon dophilus (mainly comprised of *Lactobacillus* and *Bifidobacterium*) also leads to the reduction of irinotecan-induced severe diarrhea [76]. Mechanistically, probiotic administration, including *B*. *breve*, decreases the incidence of chemotherapy-induced febrile episodes and diarrhea by enhancing SCFA production and maintaining a favorable gut microbiome [79]. Collectively, clinical evidence has demonstrated that probiotics could improve the intestinal microenvironment and prevent the adverse effects associated with chemotherapy.

### Gut microbiome in immunotherapy

In the last decade, immunotherapy has been rapidly becoming a major treatment modality for multiple types of solid cancers, including a subset of CRC. Two immune checkpoint inhibitors (ICIs), pembrolizumab and nivolumab, which are antibodies blocking programmed cell death-1 (PD-1), received regulatory approval in 2017 for the treatment of metastatic CRC that is mismatch-repair-deficient or has high levels of microsatellite instability [80]. A direct influence of gut bacteria on the efficacy of anti-PD-1 treatment for CRC has been demonstrated, suggesting that bacteria-mediated interactions with the host immune system are essential for optimal drug efficacy. Primarily activated IFNγ^+^CD8^+^ T cells and memory cells differentiated from conventional CD8^+^ T cells have a crucial role in antitumor immunity, affecting ICI therapies. In antibiotic-treated or germ-free mice, the frequency and number of intestinal IFNγ^+^CD8^+^T cells were markedly decreased, suggesting that there are specific members of the microbiome promoting their accumulation in the intestine. Furthermore, a consortium of 11 bacterial strains identified and isolated from feces of healthy human donors shows a specific induction effect on IFNγ^+^CD8^+^ T cells via the CD103^+^ dendritic cells and the major histocompatibility class IA-dependent pathway. At the functional level, colonization of these 11 strains could enhance ICI efficacy in the subcutaneous CRC mouse model with increased levels of granzyme B^+^IFNγ^+^CD8^+^ T cells and tumor-infiltrating dendritic cells [81]. Interestingly, based on this study, Vedanta Biosciences is developing a patented clinical candidate drug for enhancing the host antitumor immune response, named VE800. VE800 has been started as a first-in-patient clinical trial in combination with another anti-PD-1 ICI nivolumab to treat selected types of advanced or metastatic cancer. In addition, a recent metagenomic study on the fecal samples of patients with gastrointestinal cancer receiving anti-PD-1 treatment revealed the enrichment of *Akkermansia muciniphila*, *E. rectale*, *Lactobacillus*, and *Streptococcus* as well as the depletion of *Bacteroides* in responders [82]. These microbes may therefore be used as adjuvants for ICIs to improve cancer patients’ response to immunotherapy [83]. Consistently, the species enriched in responders are capable of producing SCFAs (*E. rectale* and *Streptococcus*), which may provide additional evidence of the crosstalk between the gut microbiome and host antitumor immunity.

Given that wide-ranging enteric microbes play key roles in ICI treatments, elucidating that the association between specific taxa and clinical response is crucial for better mechanistic insights. A recent study has revealed that *Bifidobacterium pseudolongum* enhances immunotherapy response in CRC mice through the production of metabolite inosine. The systemic translocation of inosine induced by immunotherapy activates antitumor T cells through the type 2a adenosine receptor (A_2A_R) [84]. Oral and systemic administration of inosine can improve the efficacy of ICIs that target the immune checkpoint cytotoxic T lymphocyte-associated protein 4 (CTLA-4)*,* thus reducing tumor weights and enhancing antitumor immunity. However, the effect of inosine on promoting anti-CTLA-4 therapy response is context-dependent, relying on the existence of IFNγ. Owing to the more druggable ability of small molecular agents than microbes, this study is meaningful for developing adjuvants to improve immunotherapy efficacy. On the contrary, *H. pylori*, a famous pathogen colonizing the gastric mucosa, contributes to the development of gastric cancer. A recent study has revealed that *H. pylori* infection decreases the effectiveness of cancer immunotherapies. In a mouse xenograft model using MC38 colon adenocarcinoma cells, *H. pylori*-infected mice showed significantly larger tumor volume compared to uninfected mice upon anti-CTLA-4 treatment. Mechanistically, *H. pylori* reduces the efficacy of cancer immunotherapies through deactivating dendritic cells and reducing the number and activation status of tumor-specific CD8^+^ T cells. In line with this preclinical data, two independent cohorts also showed that the efficacy of anti-PD-1 immunotherapies is lower in *H. pylori*-seropositive patients with non-small cell lung cancer [85].

Recently, the first human clinical trial validated that modulation of the gut microbiome can restore sensitivity to ICIs in patients with melanoma cancer. Ten melanoma patients who previously had no response to immunotherapy were treated with antibiotics followed by a combined treatment of FMT from two donors who exhibited a complete response to nivolumab (regression of cancer). One of the patients showed a complete response, and the other two showed partial responses after FMT. Importantly, increased expression of immune-related genes and infiltration of CD8^+^ T cells and CD68^+^ cells (antigen-presenting cells) in the gut lamina propria and tumor microenvironment were observed in these three responders. This clinical study thus provides direct evidence for the link between the gut microbiome and immunotherapy efficacy [86]. Developing a strategy to modulate the gut microbiome as an adjuvant of immunotherapy is expected to improve clinical outcomes and eventually benefit CRC patients.

## Conclusion and future perspectives

It is now clear that human microbiome is an indispensable part in the initiation, development, and progression of cancer. Emerging evidence suggests that gut microbiome confers susceptibility to CRC, directly interacts with tumors, or modulates patients’ response to chemotherapeutic drugs and immunotherapeutic agents. Although there have been promising studies that support the use of gut microbes as diagnostic markers for CRC, multiple challenges exist and impede the clinical translation of such basic findings. Firstly, the patterns of microbiome dysbiosis vary among studies and are readily affected by a combination of intrinsic and extrinsic factors, including genetic background, geographical location, diet, and medication. It remains to be explored how these environmental variables contribute to the difference in the abundance of bacteria. It has also been reported that results of microbiome sequencing are more varied among technical protocols than among populations [87]. Distinct sampling methods in the initial stage of microbiome profiling could cause variations among different studies. For instance, contamination and unsuitable storing of samples can change the composition of the fecal microbiome. Fecal samples are typically frozen and stored at −80°C; however, the conditions for storing specimens of large-scale studies are difficult to fulfill. Standardized and optimized experimental protocols are therefore crucial for sample collection, processing, and storage. Furthermore, current metagenomic-based microbial biomarkers of CRC may lead to confusing diagnostic results, because the genetic content of different microbial strains, even those belonging to the same species, may differ from 5% to 30% [88].

Characterizing gut microbes at the strain level via deep metagenomic sequencing and efficient data analysis is necessary for future translation of microbial markers. However, the cost of sequencing at increased depth could be unaffordable for many labs [89], and current bioinformatics tools may be incapable of dissecting the complexity of metagenome, demonstrating limitations such as low sequencing coverage and inferior reproducibility. Improved algorithms and analysis strategies, along with significant cost reduction in metagenomic sequencing, can potentially accelerate the development of microbial markers [90]. Similarly, large-scale studies involving particular populations may generate extensive metagenomic data for the analysis and identification of biomarkers. For example, a family-paired sampling design could partially eliminate the effects of external factors, including diets, living conditions, and activity areas [91]. A combination of genetic, transcriptional, proteomic, and metabolic characteristics, as well as the abundance level, of microbial communities may also help distinguishing CRC cases from healthy individuals with high sensitivity and specificity [53]. What’s more, longitudinal tracking of these information would yield valuable insights into the dynamics of progression in different stages of CRC, which may potentially identify better predictive biomarkers of CRC progression and outcome.

Meanwhile, although next-generation sequencing (NGS) is the most commonly used approach in microbiome studies, it has several limitations, particularly in *de novo* genome assembly. NGS generates highly fragmented and short sequencing reads, whereas microbial genomes can contain hypervariable sequences and repeating regions; thus, short reads may be inadequate for accurate genome assembly [92]. Several publications have now used novel technologies that can produce longer sequencing reads, including Pacific Biosciences (PacBio) and Oxford Nanopore technologies (ONT), to study the gut microbiome. For instance, Thidathip et al*.* adopted an ONT sequencer to profile the gut metagenome of patients with head and neck cancer [93]. Li et al. also used the third-generation sequencing technology to identify a new conditional pathogen *Enterococcus tongjius* [94]. Together, the constant advance in sequencing technology can definitely increase the understanding of the gut microbiome at higher resolution.

Given that the relationship between the gut microbiome and CRC therapy has been demonstrated in numerous studies, an unprecedented opportunity is presented to explore new clinical applications of the gut microbiome in predicting and modulating CRC therapy efficacy. Measurement of specific bacterial species in responders and non-responders may potentially predict the effectiveness of therapies in cancer patients, meriting a personalized approach to treating CRC. However, the current proof-of-concept studies have a relatively small sample size, and thus data collected from studies with a large sample size are needed to draw reliable conclusions. In addition, current species-based metagenomic studies are not detailed enough to understand which specific strains influence CRC treatment efficacy; therefore, further multi-omics and culture-dependent analytical methods are required to identify the relevant strains. Furthermore, a majority of studies are observational, lacking mechanistic insights, which are essential for understanding the interactions between specific bacterial species and cancer therapies. Great efforts should be taken to dissect the gut microbiome and reveal the intricate compositional changes associated with CRC, thereby elucidating the interactions among gut bacteria, cancer immunity, and treatment. Research methods, such as developing different antibiotics to manipulate the gut microbiome and establishing a mono-colonized mice model to validate the effect of specific bacterial strain on CRC treatment efficacy, may provide mechanistic findings for clinical translation. As we gain more knowledge on the functions of gut microbiome in cancers, we can develop new therapeutic methods based on the abundance and activity of distinct bacterial species.

## Competing interests

The authors have declared no competing interests.

## CRediT authorship contribution statement

**Yali Liu:** Investigation, Resources, Writing – original draft, Visualization. **Harry Cheuk-Hay Lau:** Writing – review & editing. **Wing Yin Cheng:** Writing – review & editing. **Jun Yu:** Writing – review & editing, Visualization, Supervision. All authors have read and approved the final manuscript.
